# The clinical and neuropsychological profiles of Alzheimer’s disease with white matter hyperintensity in North China

**DOI:** 10.3389/fneur.2024.1436030

**Published:** 2024-10-02

**Authors:** Yuan Chen, Yan Wang, Miao Zhang, Yuying Zhou, Huihong Zhang, Pan Li, Jialing Wu

**Affiliations:** ^1^Clinical College of Neurology, Neurosurgery and Neurorehabilitation, Tianjin Medical University, Tianjin, China; ^2^Department of Neurology, Tianjin Huanhu Hospital, Tianjin, China; ^3^Department of Neurology, Tianjin Huanhu Hospital Affiliated to Tianjin Medical University, Tianjin Huanhu Hospital Affiliated to Nankai University, Tianjin University Huanhu Hospital, Tianjin, China; ^4^Tianjin Key Laboratory of Cerebral Vascular and Neurodegenerative Diseases, Tianjin Neurosurgery Institute, Tianjin Huanhu Hospital, Tianjin, China

**Keywords:** Alzheimer’s disease, white matter hyperintensity, cognitive impairment, neuropsychological assessment, vascular factors

## Abstract

**Background:**

Patients with Alzheimer’s disease (AD) often exhibit characteristic clinical manifestations, particularly neuropsychiatric symptoms. Previous studies have shown that white matter hyperintensity (WMH) is strongly associated with AD progression, as well as neuropsychiatric symptoms. The purpose of this study was to investigate the clinical and neuropsychological characteristics of AD patients with WMH.

**Methods:**

This retrospective study involved 104 18-fluorodeoxyglucose-positron emission computed tomography (^18^FDG-PET-CT)-defined AD patients treated at Tianjin Huanhu Hospital from January 2010 to December 2022. Cranial magnetic resonance imaging (MRI) provided semi-quantitative data on brain structure and WMH. Collect and analyze patient clinical data. Neuropsychological assessments were used to evaluate cognitive function and psychobehavioral traits.

**Results:**

Among the 104 patients, 66 were in the WMH group (63.5%) and 38 in the non-white matter hyperintensity (non-WMH) group (36.5%). There were no significant differences in gender, age, age of onset, education, BMI, smoking, drinking, diabetes, coronary heart disease, dementia family history, fasting blood glucose, total cholesterol (TC), triglyceride (TG), high-density lipoprotein cholesterol (HDL-C), low-density lipoprotein cholesterol (LDL-C) between the two groups. The WMH group showed higher rates of hypertension, homocysteine (Hcy) levels, NPI, and CDR scores as compared to the non-WMH group (*p* < 0.05). MMSE and MoCA scores were significantly lower in the WMH group (*p* < 0.05). In the MMSE subitem analysis, patients in the WMH group showed a decrease in attention, recall, and language scores. In the MOCA subitem analysis, WMH patients had lower scores in executive function, naming, attention, language, abstraction, and orientation (*p* < 0.05). Furthermore, subgroup analysis of NPI showed a higher incidence of delusions, depression, and apathy in the WMH group (*p* < 0.05). According to the hierarchical analysis of mild, moderate and severe dementia groups, the hypertension, leukoencephalopathy, Hcy level, Fazekas total score, PWMH and DWMH scores in the severe dementia group were significantly higher than those in the mild and moderate dementia groups (*p* < 0.05). As the disease progresses, more and more patients show increased white matter hyperintensity.

**Conclusion:**

White matter lesions are closely correlated with cognitive decline and psychobehavioral symptoms in AD patients, and may be used as an indicator of disease progression. Priority should be given to early screening and prevention of WMH-related risk factors.

## Introduction

Alzheimer’s disease (AD) is a neurodegenerative disease characterized by progressive memory decline, with gradual cognitive impairment in other areas, neuropsychiatric symptoms, and personality changes as the disease progresses. Currently, the etiology of AD remains incompletely understood, but there is evidence of an association between focal white matter hypersignaling (WMHs) and clinical features of AD and disease progression (1). In recent years, a large number of neuroimaging investigations have revealed that in addition to neuronal structural damage, white matter degeneration and demyelination may also constitute significant pathophysiological characteristics (2, 3).

WMH captured by brain magnetic resonance imaging technology can be divided into periventricular high white matter signal (PWMH) and deep white matter high signal (DWMH) according to their different distribution locations, both of which have their characteristic pathophysiological pathogenesis (4). Among them, the former mainly occurred in the proximal lateral ventricle, and the latter occurred in the subcortical white matter area. Studies have shown that PWMH is thought to be caused by endothelial destruction and interstitial edema in the vicinity of the ventricle (5, 6), while DWMH originates from demyelination, gliosis, and axon cleavage (7, 8). There is evidence that vascular changes observed in Alzheimer’s disease primarily involve microvessels and may be associated with cognitive dysfunction and abnormal behavioral symptoms. Vascular factors, especially hypertension, have been identified as crucial risk elements associated with the development of WMH (9). However, the exact interaction, between vascular factors, WMH, pathology and AD severity remains an area that requires further investigation.

In this context, our study aims to preliminarily investigate the clinical and neuropsychological characteristics of patients with AD combined with WMH. By stratifying and assessing WMH classifications among the included AD patients, our objective was to provide nuanced insights into the potential impact of WMH on cognitive ability and neuropsychiatric manifestations within the spectrum of AD. This exploration is expected to shed light on the complex relationship between WMH, vascular factors and the multiple clinical manifestations of AD, thereby contributing to a more comprehensive understanding of the pathophysiological processes of the disease, as well as potential therapeutic intervention pathways.

## Materials and methods

### Subjects and inclusion criteria

This cross-sectional study was conducted by the Dementia Research Institute (DRG) at Tianjin Huanhu Hospital between 2012 and 2022. A cohort of 108 consecutive registered outpatients fulfilled the criteria for probable AD dementia according to the National Institute on Aging Alzheimer’s Association (NIA-AA) (10). All enrolled patients completed a standardized research battery of validated tests and multisequence imaging by a 3.0-T MRI scanner (MAGNETOM ESSENZA, Siemens Healthineers, Germany and Signa HDxt, GE Healthcare, United States) and by ^18^fluorodeoxyglucose-positron emission computed tomography (^18^FDG-PET-CT) (11). All of them were assessed by at least 2 experienced specialists in the field of dementia. Exclusion criteria: patients meeting any of the following criteria were excluded: (1) presence of alternative dementia types or underlying neurological or severe systemic diseases; (2) head MRI revealing white matter hyperintensity due to various causes; (3) documented history of cerebrovascular disease with a Hachinski Ischemic Score (HIS) >4; (4) history of psychiatric illness, drug abuse, or alcohol abuse; (5) severe visual or hearing impairments hindering neuropsychological examinations; (6) biomarker results indicative of other neurodegenerative diseases.

### Ethical considerations

All the subjects were accompanied by reliable caregivers, and the subjects and their families signed the informed consent form. All procedures are carried out according to the ethical standards specified by Tianjin Human trial Committee and approved [(Jin Huan) No. (2024-008)] by Ethics Committee of Tianjin Huanhu Hospital.

### Clinical evaluation and procedures

Patients’ general information, encompassing gender, age, age of onset, education level, body mass index (BMI), smoking and alcohol history, family dementia history, and medical records of conditions like hypertension, coronary heart disease, diabetes, head trauma, and surgeries was documented. Neuropsychological assessments: trained neurologists, blinded to clinical and imaging results, conducted standardized evaluations. Cognitive function was evaluated using the Mini-Mental State Examination (MMSE) (12) and Montreal Cognitive Assessment (MoCA) (13). Behavioral symptoms were assessed using the Neuropsychiatric Inventory (NPI) (14). The Clinical Dementia Rating (CDR) (15) measures the severity of dementia. The Activities of Daily Living (ADL) (16) and the Hamilton Depression Scale (HAMD-21) (17) measure daily functioning and emotional status. The MMSE scale includes 11 test items in 6 cognitive areas: attention and numeracy, memory, orientation, recall, language and visuospatial. MMSE scores of 21–26 were classified as mild dementia, 10–20 as moderate dementia, and <10 as severe dementia. The MoCA scale includes 11 test items in eight cognitive areas, including executive function, attention and concentration, memory ability, language, visual structure skills, abstract thinking, computation, and orientation. MoCA score <26 indicates cognitive dysfunction. For patients with ≤12 years of education, an assessment score of +1 was used to adjust for the effect of education (18).

### Neuroimaging and biochemical assessment

Head MRI scanning: using German Seimens Skyra 3.0 T MRI and a 20-channel head coil, the scanning included coronal, transverse and sagittal plane, and the sequences included T1WI, T2WI, FLAIR and DWI. The semi-quantitative visual score of white matter lesions was based on the Fazekas score on FLAIR imaging (range from 0 to 6). The PWMH and DWMH were evaluated blind without the radiologist’s knowledge of the clinical data (range from 0 to 3). PWMH: 0 indicates no lesion, 1 indicates hat-shaped or pencil-like thin-layer focal lesion, 2 indicates smooth halo and 3 indicates diffuse involvement of the whole anterior horn, posterior horn and body of the lateral ventricle, with or without U-shaped fiber. DWMH: 0 indicates no lesion, 1 indicates speckled lesion, 2 indicates the lesion began to fuse, and 3 indicates the lesion large area irregular fusion. The sum of the two scores is the total score. 0 indicates no white matter lesions, 1–2 indicates mild white matter lesions, 3–4 indicates moderate white matter lesions, and 5–6 indicates severe white matter lesions. Meanwhile, another senior radiologist reviewed the results to ensure objectivity and accuracy (19). This approach standardized the assessment of white matter lesions across the cohort, as well as reducing subjective bias.

The blood collection regimen consisted of an overnight fast beginning at 8 p.m., and the extraction of elbow venous blood (4–5 mL) on the next morning to analyze fasting glucose, total cholesterol (TC), triglycerides (TG), high-density lipoprotein cholesterol (HDL-C), low-density lipoprotein cholesterol (LDL-C), and homocysteine (Hcy).

### Statistical analysis

Statistical analyses were performed using SPSS22.0 (SPSS, Inc., United States). Normally distributed quantitative variables were analyzed with two independent sample *t*-tests or one-way analysis of variance (ANOVA) and were presented as mean ± standard deviation (mean ± SE). For continuous data with non-normal distribution, the data were reported as median and quartile. Between-group comparisons were conducted using the Wilcoxon Mann–Whitney test for two groups and the Kruskal–Wallis rank sum test for multiple groups. Categorical data were expressed as relative ratios (%) or percentages (%) and were analyzed using the chi-square test or Fisher’s exact test. In order to control the increase in the probability of Class I errors in multiple comparisons, we also adopted Bonferroni correction to control the error rate more reasonably and ensure the reliability and validity of the research results. A significance level of *p*-values <0.05 was considered statistically significant.

## Results

### Demographic characteristics of the study population

This study enrolled a total of 104 AD patients who met the criteria, categorized into non-WMH and WMH groups based on Fazekas scores. The non-WMH group [(38 individuals, 22 males, 16 females), average age (62.52 ± 6.04 years)] and the WMH group [(66 patients, 27 males, 39 females), average age (64.49 ± 7.36 years)] showed no significant differences in gender, age, age of onset, education, BMI, smoking, alcohol history, diabetes, coronary heart disease, family history of dementia, fasting blood glucose, TC, TG, HDL-C, LDL-C, ADL, and HAMD-21 scores (*p* > 0.05). In comparison with the non-WMH group, the WMH group had higher incidence of hypertension (34.2% vs. 57.6%, *p* = 0.022), increased Hcy level (*p* = 0.028), elevated NPI score (*p* = 0.021), and higher CDR score (*p* = 0.020). Conversely, the WMH group exhibited significantly lower MMSE (*p* = 0.000) and MoCA scores (*p* = 0.000). Table 1 summarized the clinical features of patients in both groups.

**Table 1:** Baseline demographics and clinical characteristics of non-WMH and WMH groups.

	Non-WMH (*n* = 38)	WMH (*n* = 66)	Statistic value	*p*-value
Gender [*n* (%)]			2.792^a^	0.095
Male	22 (57.90)	27 (40.90)		
Female	16 (42.10)	39 (59.10)		
Age (mean ± SE, yrs.)	62.52 ± 6.04	64.49 ± 7.36	0.360^b^	0.720
Age of onset (mean ± SE, yrs.)	60.49 ± 5.99	60.25 ± 7.36	0.418^b^	0.677
Education (mean ± SE, yrs.)	10.89 ± 3.83	10.83 ± 3.11	0.251^b^	0.803
BMI (mean ± SE, kg/m^2^)	22.51 ± 3.49	22.72 ± 3.34	0.358^b^	0.720
Smoking [*n* (%)]	13 (34.20)	18 (27.30)	0.555^a^	0.456
Drinking [*n* (%)]	11 (45.80)	13 (19.70)		0.281
Hypertension [*n* (%)]	13 (34.20)	38 (57.60)	5.268^a^	**0.022** ^*^
Diabetes [*n* (%)]	19 (50.00)	25 (37.90)		0.228
Coronary heart disease [*n* (%)]	3 (7.90)	13 (19.70)	—	0.108
Dementia family history [*n* (%)]	13 (34.20)	19 (18.80)	—	0.564
FBG (mean ± SE, mmol/L)	5.25 ± 1.86	5.98 ± 1.28	1.813^b^	0.070
TC (mean ± SE, mmol/L)	4.08 ± 1.59	4.76 ± 1.20	1.648^b^	0.099
TG (mean ± SE, mmol/L)	1.24 ± 0.69	1.37 ± 0.45	1.374^b^	0.169
HDL-C (mean ± SE, mmol/L)	1.36 ± 0.45	1.41 ± 0.33	2.496^b^	0.454
LDL-C (mean ± SE, mmol/L)	3.33 ± 3.58	3.10 ± 0.91	1.482^b^	0.138
Hcy (mean ± SE, μmol/L)	12.37 ± 4.49	14.81 ± 5.87	2.204^b^	**0.028** ^*^
MMSE (mean ± SE, score)	17.71 ± 5.42	10.87 ± 6.64	−4.631^b^	**0.000** ^*^
MoCA (mean ± SE, score)	14.50 ± 5.67	7.05 ± 5.34	−5.149^b^	**0.000** ^*^
ADL [M (P25, P75), score]	24.50 (22.00, 31.00)	28.00 (23.00, 34.00)	1.570^c^	0.116
HAMD-21[M (P25, P75), score]	5.00 (2.00, 7.50)	5.00 (4.00, 9.00)	0.952^c^	0.341
NPI [M (P25, P75), score]	6.00 (3.00, 13.50)	11.00 (5.00, 17.00)	2.309^c^	**0.021** ^*^
CDR[M (P25, P75), score]	1.00 (0.50, 1.25)	2.00 (0.50, 2.00)	2.332^c^	**0.020** ^*^

AD, Alzheimer’s disease; ADL, Activity of Daily Living Scale; BMI, body mass index; CDR, Clinical Dementia Rating Scale; FBG, fasting blood glucose; HAMD-21, Hamilton Depression Rating Scale 21-items; Hcy, homocysteine; HDL-C, high-density lipoprotein cholesterol; LDL-C, low-density lipoprotein cholesterol; MMSE, Mini-Mental State Examination; MoCA, Montreal Cognitive Assessment; NPI, Neuropsychiatric Inventory; TC, total cholesterol; TG, triglyceride; WMH, white matter hyperintensity.

a *χ2 statistic value and analyzed with chi-square test.*

b *t statistic value and analyzed with independent sample t-tests.*

c *z* statistic value and analyzed with Wilcoxon Mann Whitney test. ^*^p < 0.05 vs. non-WMH group.Bold indicates statistically significant differences.

### Neuropsychological and neuropsychiatric analysis in the study population

The detailed cognitive status of the two types of patients is summarized in Tables 2, 3. Comparing MMSE subitem scores revealed substantial differences between the WMH and non-WMH groups. Notably, the WMH group exhibited lower scores in attention (*p* = 0.014), recall (*p* = 0.003), and language (*p* = 0.040) compared to the non-WMH group. Nevertheless, there were no significant differences between the two groups in terms of orientation and immediate memory. The MoCA subitem analysis also revealed significant differences in cognitive domains between the WMH and non-WMH groups. Compared with the non-WMH group, the WMH group showed significant declines in visuospatial ability, executive function, naming, attention, language, abstraction, and orientation (*p* = 0.000). However, delayed recall did not differ notably between the two groups (*p* > 0.05). In neuropsychiatric behavior evaluation, NPI subdomain analysis showed that the incidence of delusions (*p* = 0.009), depression (*p* = 0.002), and apathy (*p* = 0.004) were higher in the WMH group than in the non-WMH group. However, there were no statistically significant differences between the two groups in terms of other psychobehavioral domains (*p* > 0.05) (Table 4).

**Table 2:** Subitems comparison of MMSE scale between the two groups.

	Non-WMH (*n* = 38)	WMH (*n* = 66)	*Z*	*p*-value
Orientation	5.50 (3.00, 7.00)	4.00 (2.00, 6.00)	−1.664	0.100
Immediate memory	3.00 (3.00, 3.00)	3.00 (2.00, 3.00)	−1.836	0.066
Attention	2.00 (1.00, 4.00)	2.00 (0.00, 2.00)	−2.462	**0.014** ^*^
Recall	0.00 (0.00, 2.00)	0.00 (0.00, 0.00)	−2.982	**0.003** ^*^
Language	2.00 (2.00, 2.00)	2.00 (1.00, 2.00)	−2.057	**0.040** ^*^

Data are M (P25, P75); analyzed with Wilcoxon Mann Whitney tests, ^*^p < 0.05 vs. non-WML group. Bold indicates statistically significant differences.

**Table 3:** Subitems comparison of MoCA scale between the two groups.

	Non-WMH (*n* = 38)	WMH (*n* = 66)	*Z*	*p*-value
Executive function	2.00 (1.00, 4.00)	1.00 (0.00, 1.00)	−4.345	**0.000** ^*^
Naming	3.00 (1.75, 3.00)	2.00 (0.75, 2.00)	−3.663	**0.000** ^*^
Attention	5.00 (3.00, 5.25)	2.00 (1.00, 4.00)	−4.476	**0.000** ^*^
Language	1.00 (0.00, 2.00)	0.00 (0.00, 1.00)	−4.678	**0.000** ^*^
Abstraction	1.00 (0.00, 2.00)	0.00 (0.00, 0.25)	−3.741	**0.000** ^*^
Recall	0.00 (0.00, 1.00)	0.00 (0.00, 0.00)	−1.186	0.234
Orientation	3.00 (2.00, 4.00)	1.50 (1.00, 3.00)	−4.067	**0.000** ^*^

Data are M (P25, P75); analyzed with Wilcoxon Mann Whitney tests, ^*^p < 0.05 vs. non-WMH group. Bold indicates statistically significant differences.

**Table 4:** Comparison of the incidence of abnormal mental behavior between the two groups [case (%)].

	Non-WMH (*n* = 38)	WMH (*n* = 66)	*χ* ^2^	*p*-value
Delusion	1 (2.60)	14 (21.20)	—	**0.009** ^*^
Hallucination	2 (5.30)	10 (15.20)		0.129
Agitation	4 (10.50)	15 (23.10)	—	0.113
Depression	5 (13.20)	28 (42.40)	9.535	**0.002** ^*^
Anxiety	8 (21.10)	24 (34.40)	2.654	0.103
Euphory	1 (2.60)	5 (7.70)	—	0.290
Apathy	8 (19.50)	33 (50.00)	8.462	**0.004** ^*^
Disinhibition	1 (2.60)	5 (7.60)	—	0.298
Irritability	10 (26.30)	26 (39.40)	1.822	0.177
Abnormal motor behavior	7 (18.40)	17 (25.80)	0.731	0.392
Nighttime	4 (10.50)	15 (22.70)	2.404	0.121
Appetite	3 (7.90)	10 (15.20)	—	0.281

Data are *χ*^2^ value; analyzed with chi-square test, ^*^p < 0.05 vs. non-WMH group. Bold indicates statistically significant differences.

### Clinical manifestations and imaging characteristics of AD patients at different disease stages

**Figure 1:**
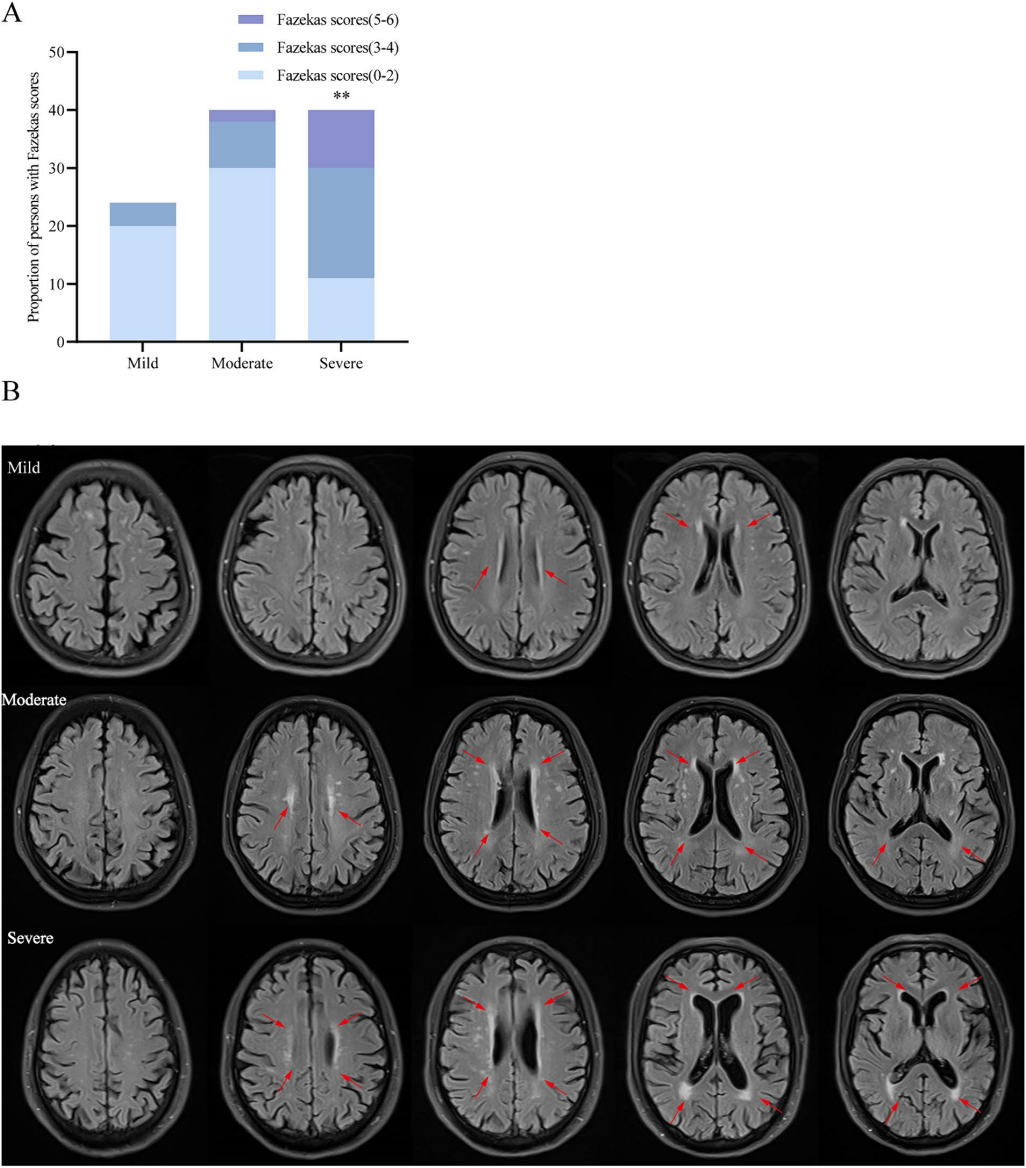
The severity of white matter lesions increases with the progression of AD. **(A)** Comparison of the proportion of total Fazekas scores among the three groups. **(B)** Brain MRI T2-FLAIR images of 3 representative cases: mild, moderate and severe. ^**^p < 0.01 vs. mild and moderate dementia group.

**Figure 2:**
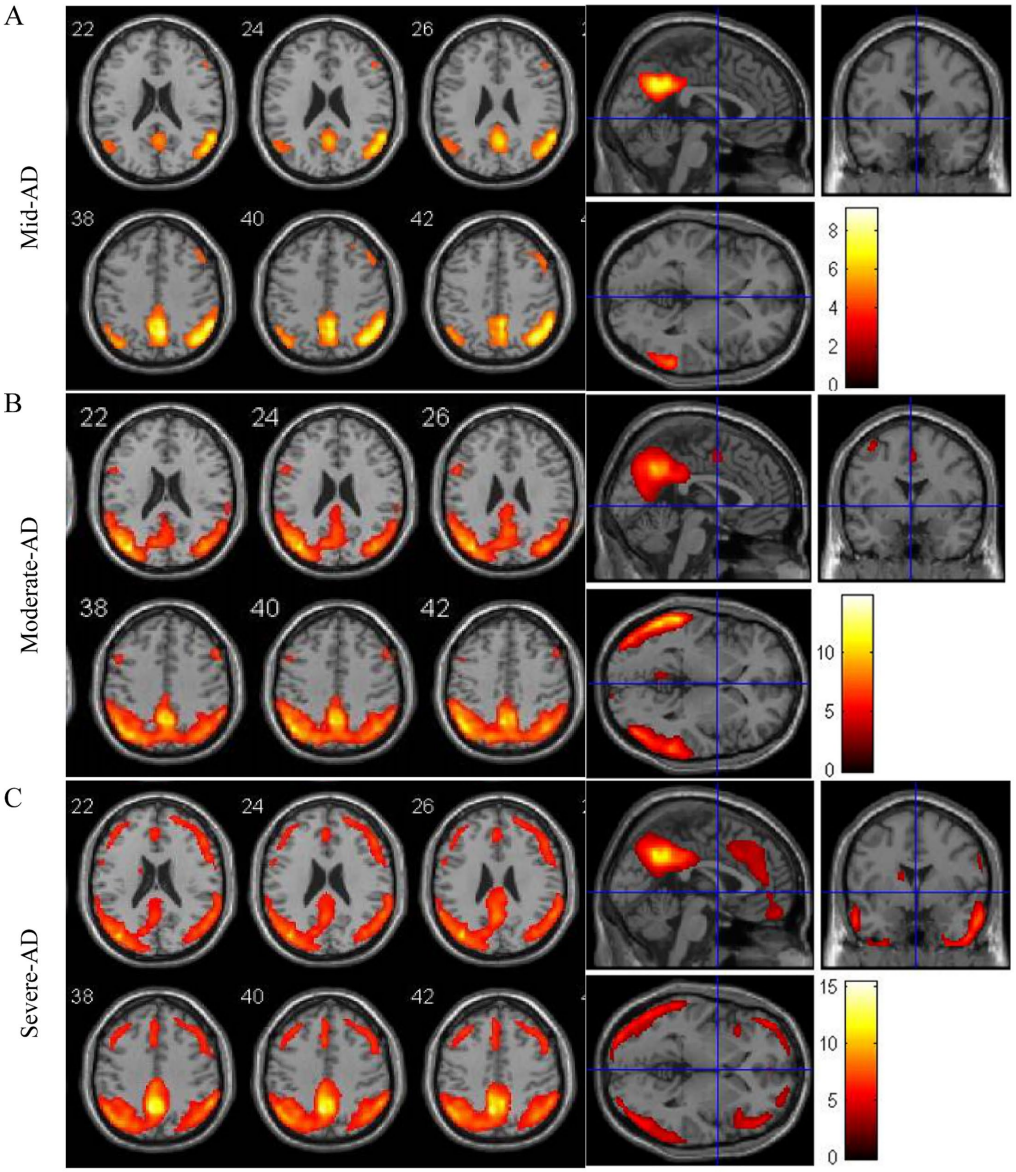
Atrophy maps of AD disease with different degrees of progression analyzed by ^18^FDG-PET-CT. **(A–C)** Typical image of AD characterized by atrophy of the temporo-parietal cortex.

To elucidate the evolution of white matter alterations during the progression of AD, we divided AD patients into three groups based on MMSE scores: mild (MMSE 21–26, 24 cases), moderate (MMSE 10–20, 40 cases), and severe dementia groups (MMSE <10, 40cases). There were no significant differences in age, age of onset, education, BMI, smoking, alcohol history, diabetes, coronary heart disease, family history of dementia, fasting blood glucose, TC, TG, HDL-C, and LDL-C among the three groups. Patients in the WMH group had a higher risk of hypertension, Hcy level, Fazekas score, PWMH score and DWMH score. Patients with severe dementia are more likely to have white matter lesions than those with mild-to-moderate dementia (Table 5). As the disease progresses, an increasing number of patients exhibit increased white matter damage, and in patients with moderate to severe cognitive impairment, MRI T2WI-FLAIR sequences clearly show multiple plaque abnormalities in the bilateral basal ganglia, semi-oval center, periventricular, and temporo-parietal regions (Figures 1A,B). Figure 2 shows the imaging features of PET-CT in different stages of AD disease. The low-metabolic brain area gradually progresses from the precuneus to the bilatemporal lobe, parieto-occipital junction cortex, and cingulate gyrus (Figures 2A,B), and finally begins to involve most frontal brain areas in the severe stage (Figure 2C).

**Table 5:** Clinical characteristics of AD patients with disease progression.

	Mild (*n* = 24)	Moderate (*n* = 40)	Severe (*n* = 40)	Statistic value	*p*-value
Gender [*n* (%)]				4.770^a^	0.092
Male	15/24	20/40	14/20		
Female	9/24	20/40	26/20		
Age (mean ± SE, yrs.)	62.21 ± 7.16	63.63 ± 7.07	64.03 ± 6.85	0.067^b^	0.967
Age of onset (mean ± SE, yrs.)	60.92 ± 7.53	60.83 ± 6.87	59.39 ± 6.65	0.554^b^	0.576
Education (mean ± SE, yrs.)	12.71 ± 3.01	10.60 ± 3.72	9.80 ± 2.84	13.589^b^	0.182
BMI (mean ± SE, kg/m^2^)	24.42 ± 3.45	22.45 ± 3.22	22.34 ± 3.44	2.634^b^	0.268
Smoking [*n* (%)]	4/24	15/40	12/40	3.113^a^	0.211
Drinking [*n* (%)]	5/24	9/40	10/40	0.159^a^	0.924
Hypertension [*n* (%)]	12/24	14/40	25/40	6.064^a^	**0.048** ^*^
Diabetes [*n* (%)]	8/24	15/40	21/40	2.873^a^	0.238
Coronary heart disease [*n* (%)]	2/24	9/40	5/40	2.728^a^	0.256
Dementia family history [*n* (%)]	12/24	9/40	11/40	5.651^a^	0.059
FBG (mean ± SE, mmol/L)	5.68 ± 2.61	5.62 ± 0.97	5.87 ± 1.13	2.709^b^	0.258
TC (mean ± SE, mmol/L)	4.12 ± 1.88	4.73 ± 1.48	4.65 ± 1.15	0.678^b^	0.712
TG (mean ± SE, mmol/L)	1.11 ± 0.57	1.37 ± 0.55	1.39 ± 0.52	3.198^b^	0.202
HDL-C (mean ± SE, mmol/L)	1.27 ± 0.47	1.49 ± 0.37	1.39 ± 0.28	3.048^b^	0.052
LDL-C (mean ± SE, mmol/L)	4.02 ± 4.36	2.93 ± 1.04	2.97 ± 0.95	1.473^b^	0.479
Hcy (mean ± SE, μmol/L)	10.32 ± 3.30	13.15 ± 4.61	16.74 ± 5.76	20.058^b^	**0.000** ^*^
WMH [*n* (%)]	9/24	22/40	35/40	18.179^a^	**0.000** ^*^
Fazekas [M (P25, P75), score]	0.00 (0.00, 3.00)	2.00 (0.00, 3.00)	3.00 (2.25, 4.75)	30.120^c^	**0.000** ^*^
PWMH Fazekas [M (P25, P75), score]	0.00 (0.00, 2.00)	1.00 (0.00, 1.00)	1.00 (1.00, 2.00)	11.553^c^	**0.003** ^*^
DWMH Fazekas [M (P25, P75), score]	0.00 (0.00, 1.00)	1.00 (0.00, 1.00)	2.00 (1.00, 2.00)	39.653^c^	**0.000** ^*^

a *χ2 statistic value and analyzed with chi-square test.*

b *t statistic value and analyzed with independent sample t-tests.*

c *z* statistic value and analyzed with Wilcoxon Mann Whitney test. ^*^p < 0.05 vs. non-WMH group.Bold indicates statistically significant differences.

## Discussion

The extracellular amyloid plaques and neurofibrillary tangles are considered to be key pathological markers in AD gray matter (20). Recent neuroimaging studies have shown that abnormalities in the micro and macro structure of white matter are associated with the risk and progression of AD. This suggests that in addition to neuronal pathology, white matter degeneration and demyelination may be crucial features in AD pathophysiology. Emerging research highlights WMH as an independent predictor of AD and is associated with decreased white matter activity in AD patients (21). In this study, 63.46% of AD patients had WMH (66/104), which was very consistent with the findings of previous studies (67.9%) (22). WMH is closely associated with cognitive decline and neuropsychiatric behavior abnormalities. The cognitive aspects are mainly manifested in the subfields of attention, recall, language and executive function, while the neuropsychiatric behaviors are mainly manifested in the subfields of delusion, depression and apathy. Of all observed factors, high blood pressure risk and high HCY level were most common in patients with WMH. Clinicians should therefore be aware of this clinical association, as early identification and treatment of these risk factors can provide new avenues for altering the course of the disease.

Age, hypertension, diabetes, hyperlipidemia, elevated homocysteine, and high sensitivity C-reactive protein are recognized risk factors for WMH, and advanced age and hypertension are key contributors (23, 24). A study by Godin et al. (25) showed that baseline blood pressure and its changes over a 4-year period were effective in predicting WMH progression in individuals aged 65 years or older. Long-term hypertension significantly affects cerebrovascular structure, increases vascular resistance, and makes the brain more susceptible to decreased blood flow and WMH (26, 27). In addition, hypertension can lead to endothelial damage, promote plasma leakage and exacerbate WMH (28, 29). Our study highlighted a notable increase in the proportion of patients with hypertension in the WMH group, suggesting the role of hypertension in WMH and AD pathology, consistent with previous findings (30). Endothelial dysfunction is closely related to the severity of WMH progression. Studies have shown that serum Hcy is associated with the degree of endothelial dysfunction and leukoencephalopathy (31). Tseng et al. (32) found that Hcy is a risk factor for stroke patients, and even a slight elevation of serum Hcy can significantly increase the severity of cerebrovascular lesions. This may be related to increased permeability of the blood-brain barrier. Yu et al. (33) reported that Hcy affected WMH severity in patients with lacunar infarction and was an independent risk factor for PWMH. Serum Hcy level in patients with acute ischemic stroke is associated not only with WMH formation, but also with the distribution of WMH. Actually, PWMH and DWMH have different pathophysiological mechanisms. The blood supply of the deep subcortical white matter is better than that of lateral ventricle, so the formation of DWMH is related to arteriolar sclerosis. The periventricular white matter is located in the junction area of arterial distribution, which is more prone to hypoperfusion injury. Therefore, PWMH formation is related to hemodynamic abnormalities. This makes the periventricular white matter more susceptible to the damage caused by elevated Hcy levels (34). Hypertension as a risk factor for WMH has also attracted widespread attention. After adjusting the influence of relevant confounders, Yang et al. (4) found that simple hypertension was not significantly correlated with PWMH severity, but was a risk factor for DWMH. This may be attributed to hyalinoid degeneration or fibrohyalinosis of small arteries caused by hypertension (35).

AD is increasingly seen as multifaceted, influenced by various factors impacting its progression and cognitive decline (36). WMH strongly correlate with worsening cognitive abilities and AD advancement (37). Simultaneous cerebrovascular issues in AD often lead to reduced cognitive performance, but the exact mechanisms remain unclear. WMH, typically caused by ischemic damage to white matter arteries, might contribute to cognitive decline and AD pathology (38). Research indicates that WMH accelerate tau protein phosphorylation, accelerating amyloid plaque and neurofibrillary tangle accumulation. This combined with Aβ-amyloid burden, affects pathways like neuroinflammation, microstructural changes, and cortical atrophy, significantly reducing cognitive reserve by disrupting vital brain networks (39). We found that the WMH group of patients significantly lower cognitive ability, particularly in attention, recall, and language. Studies have shown that WMH associated with attention, executive function and processing speed, the size and cognitive test negative correlation (40, 41). WMH location matters in cognitive decline: PWMH notably impact cognitive impairment, especially in frontal/executive function (42). In contrast, DWMH are more linked to visuospatial-executive function, language fluency, and processing speed (43). We classified AD patients according to dementia severity and found significant differences in WMH proportion, Fazekas total scores, PWMH, and DWMH scores among the mild, moderate and severe groups. Notably, DWMH scores increased with the severity of AD dementia, indicating a closer association with cognitive function in AD patients. A growing number of studies suggest a correlation between high white matter signaling in different parts of the brain and impairment in different cognitive domains. Since the anatomic location and pathological characteristics of PWMH is different from those in DWMH, studies on early cognitive dysfunction after stroke in small vascular disease have shown that the degree of DWMH is significantly correlated with attention-executive function and visuospatial function, while PWMH is significantly correlated with all cognitive functional domains (44). In this observational study, this relationship between the regional distribution of white matter damage and cognitive subdomain impairments also partially explains our findings that AD patients with WMH have significantly greater functional impairments in the subdomains of executive function, naming, attention, orientation, and abstraction than AD people without WMH. This phenomenon is especially manifested in patients with AD accompanied by DWMH, which may be related to the high risk of hypertension and high Hcy level. However, the direct pathogenicity and pathogenesis of the effects of hypertension and high Hcy level on white matter damage and cognitive subdomains in AD patients will need to be further revealed in subsequent studies.

White matter structure degeneration occurs in a variety of psychiatric conditions. There is a significant association between WMH and post-stroke depression, and higher WMH severity always indicates a greater depression risk (45). A meta-analysis highlighted severe WMH, brain atrophy, especially DWMH, as risks for depressive episodes (46). Our analysis shows that WMH group had a significantly increased rate of delusion, depression and apathy. Vascular factors not only affect the progression of WMH, but also are associated with psychiatric symptoms in AD patients. Palmqvist et al. (47) found that enlarged perivascular space in the basal ganglia area increases the risk of delusions. A recent study have shown that WMH volume in the left occipital lobe may be related to the development of delusional AD patients, and the total WMH volume and PWMH volume in the whole brain may affect the severity of delusional symptoms. A study from France in a dementia population showed that depressive mood was associated with DWMH volume (48). Hahn et al. (49) found that the occurrence of apathy symptoms in AD patients was associated with the destruction of white matter integrity. Our findings also support these views. The neuropsychiatric behavior abnormalities caused by white matter lesions may be related to the disruption of neural network connections from the frontal lobe to the basal ganglia and the cingulate gyrus (50).

Our rigorous criteria based on detailed clinical examination, neuropsychological, and multimodal neuroimaging with FDGPET, 3.0 T structural MRI, and PiB PET amyloid assays, which allowed us to reveal the neurodegenerative features of AD disease by investigating the relationship between neuroanatomical location of WMH and neuropsychological, neuropsychiatric manifestations. There are also some limitations. First, this is a case-selective clinical retrospective cross-sectional study, and the record of clinical data is inevitably incomplete, although we often make a decision with two experienced neurologists, sometimes the subjective judgment of clinicians is inevitable. The study relied on simple comparisons, which limited the evaluations of potential interactions between clinical variables and possible confounding effects. In addition, our study population included people from other parts of North China who came to the memory clinic except Tianjin, so it is necessary to expand the sample from the multi-center and multi-ethnic population to objectively reflect the disease characteristics of AD patients in North China. Second, further quantitative analysis of the image structure is needed to assess the correlation between WMH severity, WMH distribution in different brain areas and cognitive ability, psychobehavioral symptoms. Furthermore, based on this research, we will establish a causal relationship between WMH and the clinical manifestations of AD patients and explore its mechanism through a longitudinal study of a large sample.

## Conclusion

White matter lesions closely correlated with cognitive functionality and behavioral abnormalities in AD, potentially serving as markers for observing its progression. We performed a detailed clinical, neuropsychological, and neuroimaging analyses of AD patients with and without WMH. We identified several differences and, most importantly, different cognitive subdomain clinical features in disease progression. It is found that vascular factors such as hypertension and Hcy changes play an important role in AD and white matter injury, and are closely related to the progression of AD. These results provide an important clinical basis for early screening and prevention of AD patients.

## Data Availability

The original contributions presented in the study are included in the article/supplementary material, further inquiries can be directed to the corresponding authors.
